# ASO Author Reflections: Association of Hospital Market Competition with Outcomes of Complex Cancer Surgery

**DOI:** 10.1245/s10434-024-15330-9

**Published:** 2024-04-24

**Authors:** Muhammad Musaab Munir, Timothy M. Pawlik

**Affiliations:** Department of Surgery, The Ohio State University, Wexner Medical Center and James Comprehensive Cancer Center, Columbus, OH USA

## Past

Regionalization of complex surgical care stems largely from the documented improvement in clinical outcomes attributed to the volume–outcome relationship.^1^ This shift has been endorsed by numerous policy researchers, who posit that integrating health care systems enhances the capacity of clinical professionals to deliver coordinated, interdisciplinary patient care. Detractors of health care system consolidation argue, however, that a decrease in competition creates a seller’s market, granting hospitals the ability to control prices and consequently restricting patient options for care.^2^ In turn, recent work examining the relationship between hospital competition and perioperative outcomes has generated conflicting results.^3,4^ As such, the current study sought to define whether hospital market competition was associated with surgical quality and cost of care among patients receiving complex cancer care within a national dataset.^5^

## Present

Among 117,641 beneficiaries who underwent complex oncologic surgery, the mean age was 73.8 ± 6.1 years, and approximately half of the cohort was male (*n* = 56,243, 47.8 %). Overall, 63.8 % (*n* = 75,041) of the patients underwent care within a high-competition market. Notably, there was marked geographic variation relative to market competition. Hospitals in high- versus low-competition markets were more likely to be in high social vulnerability areas (35.1 % vs. 27.5 %; *p* < 0.001) and to provide care for racial/ethnic minority individuals (13.8 % vs. 7.7 %; *p* < 0.001) and patients with more comorbidities (≥ 2 Elixhauser comorbidities: 63.1 % vs. 61.1 %; *p* < 0.001). In multivariable analysis, treatment at hospitals in high- versus low-competition markets was associated with lower odds for achievement of a textbook outcome (odds ratio [OR], 0.95; 95 % confidence interval [CI] 0.91–0.99; *p* = 0.009). Patients at high-competition hospitals had greater mean index hospitalization costs ($19,462.2 [SD 16211.9] vs. $18,844.7 [SD 14994.7]) and 90-day post-discharge costs ($7,807.8 [SD 15431.3] vs. $7,332.8 [SD 14038.2]) (both *p* < 0.001) than individuals at low-competition hospitals. These data highlight the finding that hospital market competition was associated with poor achievement of an optimal postoperative outcome and greater hospitalization costs.

## Future

Results from the current study demonstrate the importance of focusing quality improvement efforts on highly competitive hospital markets to improve the delivery of cost-efficient, high-quality oncologic surgical care. For patients with cancer, grasping these dynamics can aid in making informed decisions when selecting a surgeon. Specifically, the impact of hospital competition on determining the optimal location for receiving complex oncologic care varies.^6^ Policymakers may use these findings to shape and improve financial and health policies concerning hospital competition. Moreover, these findings underscore the need to prioritize patient rehabilitation, upgrade hospital resources, and engage underprivileged communities in efforts to achieve equitable health care given the heightened vulnerability and risk for a “double disparity hit” at racial/ethnic minorities on receiving complex cancer care at high-competition hospitals due to their background and residence in socially vulnerable areas. Overall, efforts should be directed toward addressing competition-related variation in surgical care to improve equity in cancer-related treatment.
